# Veterans’ Perspectives on Interventions to Improve Retention in HIV Care

**DOI:** 10.1371/journal.pone.0148163

**Published:** 2016-02-01

**Authors:** Sophie G. Minick, Crystal L. Stafford, Barbara L. Kertz, Jeffery A. Cully, Melinda A. Stanley, Jessica A. Davila, Bich N. Dang, Maria C. Rodriguez-Barradas, Thomas P. Giordano

**Affiliations:** 1 Houston Center for Innovations in Quality, Safety and Effectiveness, Michael E. DeBakey VA Medical Center, Baylor College of Medicine, Houston, TX, United States of America; 2 Infectious Disease Section, Michael E. DeBakey VA Medical Center, Houston, TX, United States of America; Rutgers University, UNITED STATES

## Abstract

Poor retention in HIV medical care is associated with increased mortality among patients with HIV/AIDS. Developing new interventions to improve retention in HIV primary care is needed. The Department of Veteran Affairs (VA) is the largest single provider of HIV care in the US. We sought to understand what veterans would want in an intervention to improve retention in VA HIV care. We conducted 18 one-on-one interviews and 15 outpatient focus groups with 46 patients living with HIV infection from the Michael E. DeBakey VAMC (MEDVAMC). Analysis identified three focus areas for improving retention in care: developing an HIV friendly clinic environment, providing mental health and substance use treatment concurrent with HIV care and encouraging peer support from other Veterans with HIV.

## Introduction

Retention in HIV care is essential for improving outcomes for persons living with HIV. Regular visits for HIV care predict greater improvements in CD4 cell counts, higher rates of HIV viral load suppression and improved survival [1–3]. Regular HIV care allows for early detection of and intervention for poor medication adherence and behaviors that could transmit HIV to others. Retention in HIV care also supports more appropriate screening for and management of other medical conditions and comorbidities[4].

Unfortunately, retention in care in developed countries including the United States is suboptimal. A recent meta-analysis estimated that only 59% of patients diagnosed with HIV were retained in HIV care over a 12-month period, [3] and CDC estimates that only 40% of persons with HIV infection in the US (including the undiagnosed) are engaged in HIV care [5]. Patients experience a variety of barriers to retention in care, such as poor or no access to care, unstable housing, substance abuse, mental illnesses, stigma and low literacy [6–9]. Observational data and uncontrolled intervention studies suggest that case management, outreach, social services and mental health services, to decrease unmet medical and social needs, can improve retention in care [10]. Brief provider and clinic staff messaging focused on promoting retention and a welcoming clinic environment improved clinic attendance in a multi-site pre/post intervention study [11]. Guidelines to improve retention in care have been developed and disseminated [12], but, because this is a relatively new field of scientific inquiry, there are few individual-level interventions proven in randomized clinical trials to improve retention in care [13]. Recommendations suggest that interventions should be further developed to assist in improving retention [12].

Retention in care represents the greatest opportunity to improve outcomes among the population of persons living with HIV infection [12, 14]. Subsequently, we conducted a qualitative study to obtain patient input on the design of interventions to improve retention in care. We conducted this study at a Department of Veterans Affairs (VA) hospital. The VA is the largest integrated healthcare system in the US and the largest single provider of care to HIV-infected patients in the US, caring for about 24,000 HIV infected patients in the US annually [15]. We sought input from patients with ranges of experience of care, including patients who are currently having difficulties being retained in care and/or have had a history of poor retention and patients who have maintained retention in care since initial diagnosis. We asked patients to reflect on their experiences since being diagnosed with HIV and importantly, to provide examples of solutions they feel would be beneficial to improve retention in care in general and specifically within the VA hospital system. Results from this study will inform an intervention to promote retention in care.

## Methods

### Participants

Participants were recruited from Houston’s Michael E. DeBakey VA Medical Center between June 2011 and September 2013. Purposeful sampling was used to gain a representative sample of the HIV community at the VA including both newly diagnosed/out-of-care hospitalized inpatients and non-hospitalized outpatients, regardless of their current retention status. Recruitment, eligibility and procedures varied slightly to accommodate the specific needs of inpatients contrasting with outpatients. Focus groups were held for patients currently in outpatient care. One-on-one directed interviews were conducted with inpatients who were hospitalized. Patients were determined ineligible due to active mental health or physical conditions impairing their ability to provide consent or meaningfully participate in the qualitative research. This study was approved by the Institutional Review Board for Baylor College of Medicine and Affiliated Hospitals; all participants gave written informed consent.

### Recruitment and Eligibility for Outpatients

Outpatients who presented to the HIV clinic for either a regularly scheduled appointment or a walk-in visit were eligible to participate in a focus group. Research coordinators attended clinic, and after obtaining permission from an eligible patient’s HIV primary physician and being introduced to the patient by their HIV primary physician, recruited all willing patients. Patients who agreed to participate in a focus group provided written informed consent at the time of recruitment and then completed a brief demographic survey. They were scheduled to return for a focus group no more than two weeks from the recruitment date. To increase focus group homogeneity, participants were invited to attend focus group sessions based on self-reported sexual orientation. We attempted to contact patients who missed their scheduled focus group and invite them to participate in the next scheduled focus group.

Due to the small number of female veterans with HIV infection, we did not expect that there would be a large enough sample of women to complete a women’s focus group. However, we wanted to ensure that the perspectives of female Veterans were captured as their experiences could be different from male Veterans’ perspectives. Therefore, all female patients who attended a clinic appointment were invited to complete a one-on-one interview.

### Recruitment and Eligibility for Inpatients

Inpatients either recently diagnosed or out of care were eligible for one-on-one interviews. “Out-of-care” was defined as patients who had no primary care visits in two or more of the previous four quarter-years, which has been previously used as a measure to identify patients with poor retention in HIV care and has been associated with increased risk of mortality in prior VA research [1]. We reviewed medical records of patients who had only two quarters with visits to confirm that the patient was not virally suppressed and purposefully seen infrequently. All inpatients diagnosed within the previous 12 months were eligible, regardless of “in care” status, because recently diagnosed are also at high risk of poor retention in care [16]. Inpatients who were expected to be discharged to hospice care, prison, or any other institutional setting were ineligible because the VA would not be responsible for their subsequent care. To identify potential participants, we communicated with the HIV clinic director and the infectious diseases consult team, who are routinely notified when any patient with HIV infection is hospitalized. Once the patient agreed to speak to the coordinator, the coordinator approached the patient for recruitment. If the patient agreed to participate, he or she provided written consent, completed a brief demographic survey and completed the interview before discharge from the hospital.

### Procedures

The brief survey completed at the time of recruitment asked patients to self-report their age, gender, race/ethnicity and sexual orientation. Participants also reported the month and year of their HIV diagnosis, date of entry into HIV care and date of entry into HIV care at the VA. All focus groups and interviews were conducted in the VA medical center. One interview guide was used to collect data regardless of recruitment strategy. Questions in the guide were organized into categories to explore patients’ perceptions and experiences of HIV care received at the VA since HIV diagnosis (guide in S1 File). Each interview and focus group session lasted up to 60 minutes and was conducted by CLS, a female public health researcher and practitioner interested in health care delivery and health care outcomes. BLK, a female Infectious Disease research coordinator interested in facilitators and barriers to HIV treatment, was present but not involved in the sessions, passively taking additional notes. Each session was audio recorded and transcribed verbatim. Further details discussing reflexivity can be found in the attached COREQ checklist (S2 File).

Participants’ electronic medical records were reviewed for the first CD4 cell count and HIV viral load laboratory values available at the VA and laboratory results current as of the interview date. Paper medical records were reviewed for results that pre-dated the electronic medical record (i.e. before about 1997). Electronic records were reviewed for clinic attendance to assign retention in-care status.

### Analysis

Frequency distributions of the demographic data were used to describe the patient population and analyzed using SAS.

To further characterize the retention in care status of the study population, various measures of retention in care were applied using HIV primary care appointment attendance data for the two years before enrollment. 1) gap in care: was there a gap between attended appointments spanning longer than 180 days during the two years?; 2) two-year retention: did the participant attend at least one visit in each of the four 6-month blocks during the two years?; and 3) one-year retention: did the participant attend at least one visit in three or four of the four quarter-years during the one year before enrollment[17–19]? Finally, to summarize the retention data, we created a single variable representing poor retention in care in which participants meeting any of the three measures of poor retention were considered to have some evidence of poor retention in the past 2 years.

Transcripts were imported into Atlas ti. version 6.2 for data management. As data were collected, content analysis was performed and themes were extracted deductively. Content analysis allows data to be summarized and categorized into themes and categories [20]. Passages of data were coded and categorized under the corresponding pre-specified three categories (barriers, facilitators and interventions for HIV care); codes within the core categories were not pre-specified. Recruitment ceased once data saturation was reached in each stratum. Two researchers, CLS and BLK developed a list of codes that corresponded with the data presented in the interviews and focus groups. The separate code lists were later compared and a code book developed using the codes that both researchers agreed upon. If passages or codes were not similarly coded both researchers, they were presented to other members of the study team and a consensus was reached. Each transcript and codes were compared and validated by CLS, BLK and SGM, a female public health researcher interested in increasing access to care for individuals with HIV. Thematic analysis proceeded to present data cohesively.

## Results

### Participant characteristics

**Fig 1:**
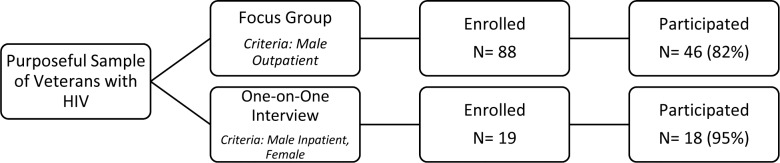
Participation flow diagram.

A total of 107 patients were enrolled, including 88 who agreed to attend a focus group and 19 patients who agreed to participate in one-on-one interviews. Forty-six veterans attended 15 different focus groups (participation rate 52%). All were male outpatients per study design. Eighteen veterans completed a one-on-one interview (participation rate 95%). This comprised of 4 female outpatients, and 14 inpatients; 12 male and 2 female (Fig 1).

Thirty-three eligible patients declined enrollment in the study, the majority declining due to not having the time that the study would request. Among enrolled participants, there were no significant demographic differences between those who participated and those who did not (data shown in S1 Table). Enrolled patients who did not actually participate in a focus group reported work, scheduling and distance from the VA as primary reasons for not attending their focus group.

Participants’ ages ranged from 27 to 79 years. The majority of the participant population was age 50 and older (88%) and diagnosed with HIV for over 10 years before enrollment (83%). About two-thirds of the patients had a gap in care over 180 days long within the last 2 years (63%), and 20% currently had a detectable HIV viral load (Table 1). Nearly three-quarters of participants (72%) met at least one definition of being out-of-care within the last two years.

**Table 1:** Demographic and clinical characteristics of participating patients (N = 64).

	Participants (N = 64)
*Age*	
<40	2 (3%)
40–49	6 (9%)
50+	56 (88%)
*Sex*	
Male	58 (91%)
Female	6 (9%)
*Race/ethnicity*	
Black	43 (67%)
Hispanic	8 (13%)
White	13 (20%)
*Sexual Orientation*	
Homosexual or gay	21 (33%)
Bisexual	6 (19%)
Heterosexual or straight	35 (55%)
Unsure/ In-transition	2 (3%)
*Years from HIV Diagnosis*	
≤5 years	5 (8%)
6–10 years	7 (11%)
11–20 years	24 (38%)
>20 years	28 (44%)
*Retention in care* *	
*No gap in care ≥ 180 days in last two years*	
Not retained	39 (63%)
Retained	23 (37%)
*Constancy in care in last 2 years (≥ 1 visit in each 6-month block)*	
Not retained	27 (44%)
Retained	34 (56%)
*Constancy in care in last 1 year (≥ 1 visit in 3 or 4 quarter-years)*	
Not retained	24 (39%)
Retained	38 (61%)
*Not retained by any of the 3 definitions above*	
Not retained	44 (72%)
Retained	17 (28%)
*First HIV Viral Load result [copies/mL]*	
≤ 400	16 (23%)
> 400	48 (78%)
*First CD4 cell count result [cells/mm*^*3*^*]*	
<200	15 (23%)
200–500	27 (42%)
> 500	22 (34%)
*Current HIV Viral Load result [copies/mL]*	
≤ 400	51 (80%)
> 400	13 (20%)
*Current CD4 cell count result [cells/mm*^*3*^*]*	
< 200	10 (16%)
200–500	29 (45%)
> 500	25 (39%)

*Veterans excluded from retention measures include: two Veterans from the gap in care measure due to only having one visit in time period; three Veterans from the two year retention measure due to either newly diagnosed or new to the VA within two years; two Veterans from the one year retention measure due to either diagnosed or new to VA within one year.

Inpatients had longer gaps in HIV care and lower current CD4 cell counts (S2 Table). Selected characteristics of participants quoted in this report are provided in S3 Table. S4 Table provides a comprehensive overview of the results. It presents the most common emergent themes with regard to patient-identified barriers, facilitators and suggested interventions for improving retention in care as they appeared throughout the interviews.

**Fig 2:**
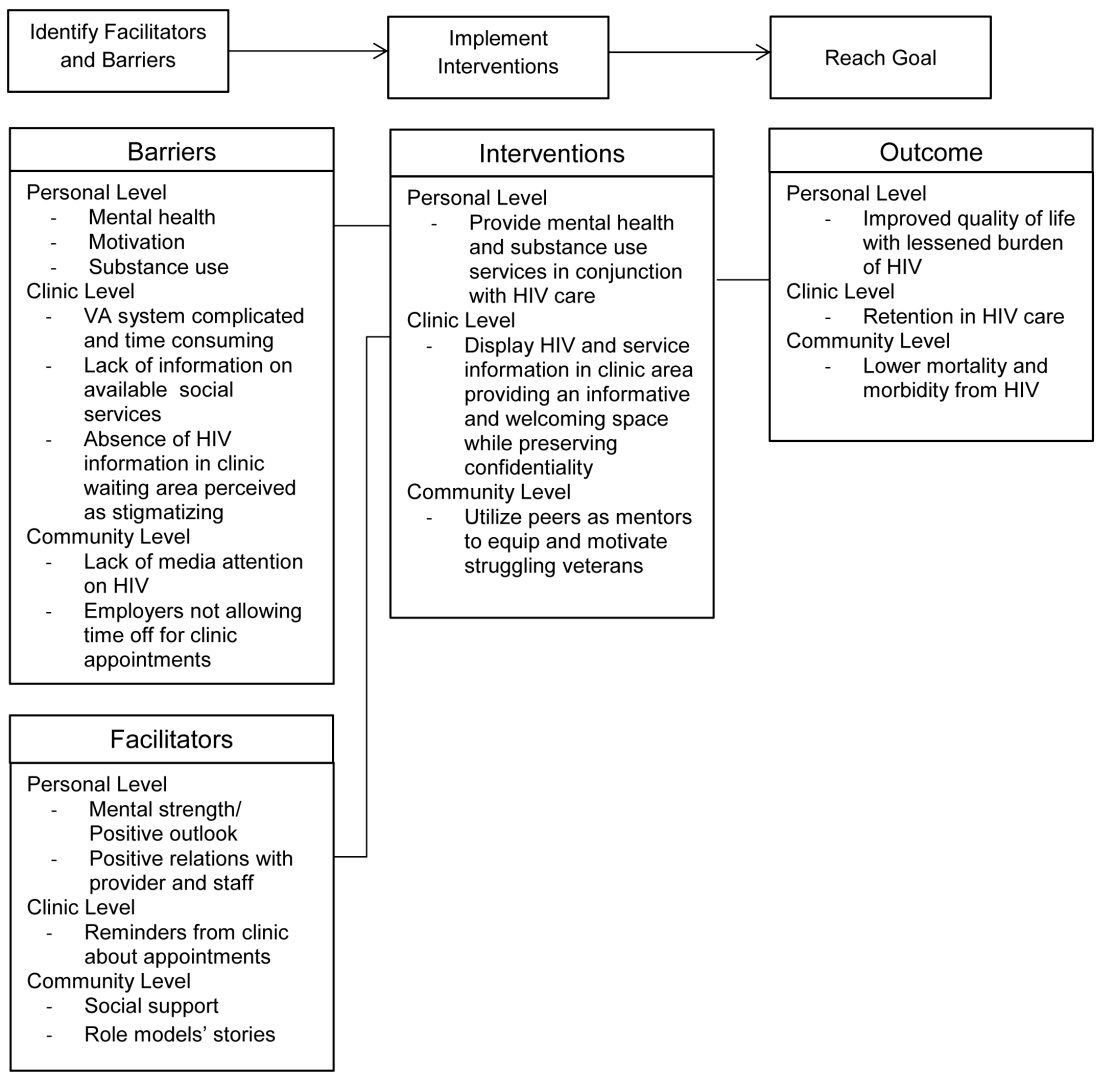
Intervention map to achieve routine care for veterans with HIV.

Fig 2 presents a cohesive intervention map that highlights the prominent findings from these results and suggests specific interventions based on these data.

### Barriers

Three categories of barriers to attending HIV outpatient appointments emerged: “My self” (the patient himself or herself), “My needs” (his or her unaddressed needs), and “My clinic” (the clinic’s characteristics) (S4 Table).

#### My Self- “I am my worst enemy.” (001)

Patients were quick to attribute poor retention in care to their own choices and mindset. In each of these instances, the patient’s psychological state determined whether or not a visit would be attempted.

*“I’m at my place and I know that I need to go… But I can’t motivate myself to go for whatever reasons I’ve got going on in my head*.*”* (002)

Often after initial feelings of hopelessness and helplessness at diagnosis, patients adopt the attitude that nothing he or she can do can fix their situation, thus no action will be taken, even among persons diagnosed relatively recently.

*“I felt like I was dying and I- and I ain’t think there was no help for me*.*” (003)*

Looking back, patients recognize these mindsets sprung from inaccurate beliefs that HIV is a death sentence rather than a manageable chronic disease. Hopelessness may reoccur well past diagnosis and continue to affect patients’ choices to keep connected with their doctors and appointments.

*“It’s not a- a time… because like me it’s been ten years and like right now I’m going through a bit of discouragement…You- it’s okay to tell them (patients with HIV) in the beginning but you know one day they might be having a moment*.*” (004)*

Patients recognize this struggle of a lifetime battle of illness and fatigue that follows. One veteran explained a difficult lesson she learned from her friend.

*“I took care of her… 18 years but after she gave up taking up her meds; she got tired of being sick*. *And uh*, *she ca- she ca- she quit taking her meds and she got pneumonia and that’s how she passed*. *She brought it on herself; she got tired of taking all that meds; so she quit taking it*.*” (003)*

Other veterans may deem a clinic visit unnecessary when things are going well.

*“And once I found that I was non-detectable*, *I didn’t think I had to continue coming to the doctor which was an*, *um*, *no-no*. *I mean you have to come so they can keep up with you constantly*.*” (005)*

"*Unfortunately when you don’t feel sick and…uh T cells are good and you’re undetectable*, *then you’re not as concerned so you tend to miss some of ‘em without*, *you know*, *concern*.*”(006)*

#### My Needs–“I need more help than I can get on my own.” (001)

Veterans reported that conflicting and unaddressed needs continue to prevent them from accessing regular care. Mental health problems, substance use, financial concerns, transportation problems, lack of social support and the burden of time away from work all contributed to difficulty staying in care.

Patients recognize that these issues play a large role in their HIV care.

*“I still have other psychological problems that come into play and I’m not always on top of my care as I should be*.*” (007)*

Participants frequently reported that substance use was a substantial barrier to retention in care. Alcohol and drugs took precedent over appointments. Patients were not equipped to handle both their substance use and their HIV infection.

*“I went through a phase of substance abuse and*, *and bein’ a drunk*. *And*, *uh that really affected*, *that was the most serious thing that ever really like affected my diligence to takin’ my medications and seein’ my doctor*.*”(008)*

*“My main thing about taking my meds is that I’m an alcoholic and when I start back drinking; I stop taking medication*. *And that’s when I start missing appointments*. *A lot of people that miss appointments is because they either still using drugs or they’re uh not in a safe place or they’re not prepared…You know I just- I uh- I cry a lot and feel embarrassed and shame doing drugs and alcohol more than I do uh living with HIV today*.*” (005)*

Commitments, such as an inflexible working schedule, made scheduling and attending laboratory and/or physician appointments a challenge, particularly for patients who could not afford to take time off from work.

*“Because I had a full time job and um I had to schedule–try to schedule my appointments around there but they had–they had so many people that it was real hard for me to schedule it to even see my doctor and that’s why I stopped worrying about my appointment*.*” (009)*

*“I do the type of work that if I’m not there I’m not gettin’ paid and that’s the thing that sorta gets to me more than anything*. *And*, *and I struggle financially to a certain degree to the point where it’s just a total loss that whole day for me when I come to the doctor*. *You know what I mean*?*” (010)*

#### My Clinic–“Hurry up and wait.” (001)

Personal barriers overlap within the structure of the clinic itself. Case managers are intended to help a patient manage these personal needs, however some patients believe case managers are too rushed to be useful.

*“If they just sit down with you instead of just passing it to you like they’re in a hurry and just kind of push you along; I think that’s–and that’s what case management is I think is supposed to sit you down and let’s figure out what you need*.*”(011)*

Patients also mentioned difficulty accessing other programs to get help with barriers to care.

*“Well*, *one thing in the past it has been pretty hard to get into a drug program here at the VA*. *Uh*, *you can’t just go walk down there and say*, *‘I have a drug problem*, *can you help me*?*’ Because the VA is a ‘hurry up and wait organization’*.*” (001)*

Many veterans found long wait times at the clinic problematic. The common expectation was that the majority of the day would be consumed by appointment visits.

*“I was sittin’ here for a good 3 hours and*, *and*, *I’m not gonna do that*. *No I’m not gonna do*.*” (012)*

*“They had too many comin’ in the mornin’*, *and too many in the afternoon*.*” (013)*

Veterans express frustrations with maneuvering the size of the Veterans Affairs hospital, from difficulty parking to finding their way around the building.

“*The VA is a big place um getting around; you know problematic feeling like um you know where to go*.*” (003)*

*“The worst thing of course is the parking*. *That discourages*. *And I meant it*.*” (014)*

Once patients make it to the outpatient clinic area, several noted that materials related to HIV and HIV care were absent. The lack of HIV information contributed to a sense of not feeling welcome in the VA.

*“And then you go to the clinic and you’re sitting there and you don’t see anything about HIV*. *(…) to me it just kind of seems like- like- like we’re still hiding HIV(…) to me it still feels that the VA is still kind of hiding the HIV stuff*,*(…) “Do they need to hide us*?*” or- or*, *“Did I do something wrong that I need to be hidden*?*” (015)*

A few patients report prior experiences with being judged by the provider and clinic staff as deterrents to wanting to attend future appointments. Amidst all the other challenges to receiving care, one bad experience or poor relationship with one’s doctor is enough to deter future visits.

*“Well*, *I don’t like coming to appointments because I’m always in trouble when I come to the appointments*. *Doctor is always on my case*.*” (001)*

*“If you get kind of a negative reaction from your health care giver*, *you kinda don’t want to go back*.*” (016)*

### Facilitators

Even with its challenges, veterans gave mostly positive reviews about receiving care at the VA. Veterans identified 4 key areas that were most helpful to continuing care: “My desire,” “My perspective,” “My community" and “My tools.”

#### My Desire–“I want to stay alive and healthy” (017)

Many patients talked about a transformative moment where their outlook changed from despair to desiring life. This shift of taking on personal responsibility for their health was reported as an important factor in coming to appointments.

“*I had to get to that point where I realized and knew that it all about bein’ there for myself*, *wanting more for me and do I*? *At times I do… I don’t find anything hard–I try- you know about staying in care other–because I care about my health*.*” (001)*

This positive mindset focused on what it means to accept life with HIV.

*“Honey*, *once they told me I know I*, *I wanted to live*. *I know that*. *So I did what I had to do in order to do that*.*” (018)*

Family was a large motivation for staying in care and taking care of one’s health. Veterans talked about the value of family and how they want to take care of themselves so they can be there for their children and grandchildren.

*“They kiss me you know and they hug me*. *We love you granny; we love you granny*. *They call me all the time*, *they and they make me feel*. *They make me want to live; go on and on*.*” (003)*

#### My Perspective- “I’m not dying of AIDS, I’m living with HIV.” (019)

Some patients moved from hopelessness to empowerment because they were shown that health was achievable. Other people living healthy, full lives with HIV infection emerged as strong role models. Inspired, patients reported they followed their examples.

*“What really inspired me*, *what really*, *really inspired me*, *when Magic Johnson came on television and announced that he was positive*. *I mean on live television*. *He came out*. *And a man of–uh*, *of his stature and if he can come out and say*, *‘Hey I got this*,*’ and have a positive attitude about it and then I could*. *So that inspired me and I started to go see the doctor more*.*” (020)*

Veterans credit other patients with HIV infection as a source of great encouragement to follow in their steps and get care. This female veteran captured the personal impact of the words of someone who has survived a similar struggle to hers.

*“When I first was diagnosed*, *I met this guy*, *I still remember him*. *And he saw my face and he said um*, *‘You just found out huh*?*’ I said yeah*. *He said*, *‘Baby I’m in- and that was ten years ago and he’s still living’*. *He said*, *‘I’ve been diagnosed for twenty years*.*’ He told me that*. *He said*, *‘The only thing I can tell you is these two words and it’s*, *“Just live*.*”‘ And I took those two words and I tell that to everybody that I meet that’s HIV positive that- that’s discouraged and don’t want to get doctors*. *(…) So I can tell you it- it’s not the end of the world*. *So it’s easy–it- it’s easier coming from a person that’s- that’s lived that’ that’s walked in those shoes than from a doctor saying*, *‘Oh come get your care*.*’” (021)*

*“You need to get someone who’s had issues who’s you know related to having an upset stomach*, *to getting headaches*, *throwing up; you need to have someone that- that knows about it*. *Not just someone who can read about it*.*” (011)*

Trust in the doctor was another important factor in patients’ decisions to attend appointments. Veterans reported strong respect for their doctors and trust in them for medical guidance.

*“I’m not going to try to make decisions; medical decisions especially for myself*. *So I just put all the decision; everything on him*. *When he said to come back I came back*.*… 20 years later I’m still here so clearly it wasn’t a mistake*.*” (022)*

#### My Community–“Everybody needs a support system.” (021)

HIV is a lifelong battle. Veterans recognize that ongoing community support, especially from one’s own family, is a critical component to health.

*“You know you just can’t–you cannot do it by yourself*. *I don’t care*, *you are not superman no–even me*.*” (021)*

*“My husband*, *he takes me to all my appointments*, *tell me where I need to go and how to get there and he be with me step by step*.*” (003)*

Veterans testify that the VA provides a thorough and unique caring atmosphere from the staff at the front desk to the providers.

*“Only thing that’s keepin’ me here is that I actually like that they care about you because if it wasn’t for that I wouldn’t be comin’ all the way over here*.*” (012)*

Not only are doctors trusted, they also invest in relationship with their patients. This relationship is a driving force for veterans to return to their visits even in spite of other difficulties.

*“I come because I like my doctor*. *I like- actually I like both of my doctors and uh they always make me feel good um when I get here and- and go out feeling good*.*” (016)*

#### My Tools–“I know my health’s gonna be taken care of… the system’s great.”(006)

The affordability of the VA was reported as a great tool for veterans who otherwise have limited healthcare options. Once they gain entry into the system, veterans report that they are secure, knowing their health will be taken care of regardless of financial status.

*“It was easy for me to be able to come to the VA and knowing that I’m you know; I’m n- I’m not worried about paying*.*” (004)*

*“I mean the VA’s doin’ all it can do*. *I mean they’re givin’ us free medicines*. *Ain’t no way we can afford this junk on our own*. *You know*?*” (020)*

Patients benefit from appointment reminders both in the mail as well as over the phone, which reportedly help.

“*That little phone call that you give us to let you know that you have an appointment is scheduled for that–that week coming up*. *That uh- that- I love that because I forget unless you- and when you call me I know*.*” (009)*

Other tools veterans found helpful include support groups, transportation and case management.

*“I think the support group was the biggest help to me*.*” (014)*

*“I was in a safe haven here at the VA*. *They you know they- they just awesome*. *Everything I could’ve ever needed uh and- and I could take care of my wants; it was there*.*” (005)*

### Interventions

Despite the facilitators reported above, retention in care is still known to be suboptimal at the VA. Veterans took some time to think about ways to improve retention in care for themselves and others. Three areas of focus arose: services, knowledge and support.

#### Services–“Let’s make you a map and get you there.” (011)

Patients suggest addressing needs as soon as they are identified. Providing mental health and substance treatment concurrent with HIV clinical care would address the major barriers for many patients.

*“I would like to see a combination of*, *of that you know*. *Drug and alcohol rehabilitation combined with HIV together*.*” (020)*

Patients report a gap between the available services and their awareness of those services. They suggest that providers be proactive in sharing different services that may be available and beneficial to them. Patients suggest implementing a service information lounge, a casual gathering place where veterans can lounge, learn, interact and grab information about HIV and HIV care.

*“Have an office; like a go-to place (…) That’s great to have somewhere like that where you can go in*, *maybe drink a cup of coffee and find out maybe some support*.*”* (023)

#### Knowledge–“A lot of people just don’t know about HIV. They think they know, but they really don’t.” (009)

Participants respond that knowledge is lacking; both about HIV in general, as well as how to navigate the VA’s system of care. Not only do they want to be more informed, they also suggest that the public could benefit from more knowledge. Participants believe education helps raise awareness and improve general health as well as decrease stigma of HIV. They suggested events or courses about HIV infection for both veterans and extended community regardless of HIV-status.

*“It (educational program offered by CDC at another location) teaches you about HIV*. *Everything about it from ins and outs*, *funding you know the goods and bads*, *um statistics; the City of Houston comes and it’s really educational*. *If we could bring some of that to like a program here and do like a nex- next step program…for veterans to take um I think it’ll be a good eye opener because people don’t know all there is to know about HIV*.*” (011)*

*“I think you could do like a- a class and actually have veterans that are going through it and um you know kind of lead the class and let the veterans take the class too and teach others*.*” (011)*

Veterans want their disease to be acknowledged. These education and public awareness campaigns were suggested as ways to also fight the stigma of HIV infection. Increasing acceptability and support in a community relieves some of the burden from an individual who would otherwise struggle alone.

*"People need to stop shying away from the subject of HIV because it’s there… it needs to be addressed more openly and stop*.*" (004)*

*“Uh*, *I rarely see anything on*, *uh television about*, *uh prevention of HIV or*, *or HIV meds*. *Uh*, *I*, *I would like to see more out in the open*, *uh people are still afraid of it*.*”(019)*

Patients are concerned about the lack of HIV information in the clinic and believe clinics should be more open about providing HIV care. They suggested that adding information to the walls in the clinic can promote a more “HIV friendly” environment at the VA to reduce HIV stigma.

*“When I look on the board out there I don’t see anything about HIV but I see everything else ya know*?*” (024)*

*“So- so maybe more- may- maybe more like more posters*, *more literature in the rooms; that way people can say*, *‘Oh okay they (the VA) are not afraid*.*’” (015)*

As many patients find the VA system difficult to navigate, participants proposed a video to help guide the patient through the hospital system with what to expect specifically. This video could be threefold, providing HIV information, VA system navigation and motivational support.

*“That video would not only explain to them* (veterans with HIV) *what was going on with them but what were the probabilities and then they would talk about all the things that helps in that area and then they’re given the rest of the information on the video*. *That’ll make them interested to come back if they know exactly what they’re going to be expecting*.*” (002)*

#### Support- “I’m not alone… you know it’s another soldier.” (004)

Veterans reported that an intervention that reduced hopelessness after diagnosis would encourage persons to be less avoidant of HIV care. The more powerful testimonies suggested that peers who have already experienced success in managing HIV would be helpful.

*“That would have really benefit me when I first found out… Someone that already had it can tell me what’s going on you know and they done been through and you know it’s nothing to fear*. *Just*, *you know*, *do this*.*” (025)“You know people seem to listen to their peers more than somebody else*.*” (026)*

Veterans comment on the special situation of being a veteran with HIV as well and the comfort of knowing that there are other people in similar situations. A veteran explained the benefit of a fellow veteran mentor.

*“You’ll say well I’m not alone… You know it’s- it’s another soldier*.*” (004)*

On a practical level, experienced peers were suggested to not only encourage hope; they provide practical direction on navigating the system of care.

*“If it’s a veteran it bridges the gap a whole lot easier just because they’re going through the same thing*. *This veteran has gone to the VA hospital and done whatever he had to do and now still looks decent*, *still looks healthy and still going strong*.*” (002)*

Peer-to-peer support does not have to be restricted to newcomers but can be utilized throughout the HIV care continuum and in larger settings such as support groups, as this long-term veteran explains.

*“Support groups are so important*. *I had no idea the support groups and then they wanted me to go into a group session which I thought was just outrageous but that is where I mean people say things and then it clicks*. *Like oh yeah and uh it is amazing the help that a support group gives you in a- in a group setting you know and not one on one which is what I thought I would want*.*” (022)*

Participants suggested alternative mediums of delivering messages that encourage retention in HIV care that would have reach and mobility beyond extensive personal contact. Ideas included videos (both navigational and motivational), clinic posters and bulletins and publicity through celebrity broadcasting, commercials, newspapers, internet or social media.

## Discussion

This study provides insight from veterans living with HIV into the barriers, facilitators and interventions to improve retention in HIV care. Veterans reported numerous obstacles and challenges to attending regular HIV clinic appointments that are similar to results from past studies [6–9]. While others have reported barriers and facilitators to care, our study uniquely reports suggestions from veterans for intervention components. An intervention that alleviates several barriers and supports numerous facilitators is desired. An intervention map based on our results is present in Fig 2.

The clinic can play a key role in creating a positive environment for patients with HIV. Veterans consistently reported that positive interactions with doctors and staff encouraged them to attend their HIV appointments. Better experiences with doctors and clinics have been associated with greater retention in HIV care [21, 22]. Veterans report that the clinic could do more to destigmatize HIV. More awareness of and information about HIV infection can help. Veterans suggest that an HIV-friendly environment providing more information about HIV and staffed with providers who deliver patient oriented services could improve retention in care.

Providing mental health and substance use treatment concurrent with HIV care was another patient-centered intervention that could improve retention in care. Mental health disorders and substance abuse are common comorbidities with HIV infection and the integration of treatment is recognized as a key challenge [23]. At the time of the study, mental health services, including specialized social work counseling and clinical psychologist services, were offered conjointly with HIV care in the Houston VA. Additional services co-located in the HIV clinic (i.e. on site substance dependence treatment and psychiatric care) could address this recommendation, but system changes and truly co-located mental health and HIV care are difficult to achieve. Better collaboration and navigation between the services could improve services and patients’ perception of close collaboration [23, 24].

The unique bonds within the veteran community can be a powerful tool to provide support for veterans living with HIV infection. Peer support is influential on multiple levels. Although everyone’s individual story is different, veterans share similar experiences and needs by virtue of their veteran and HIV status. Peer mentoring models have been implemented with varying success in VA and non-VA settings [25, 26]. The testimonies of peers’ successes are motivational and can lead to acceptance of the possibility of real change, providing hope to struggling veterans.

The VA recently introduced a Peer Support Program (PSP) in mental health and substance use clinics, in which trained veterans who have overcome mental health and substance use problems provide support for other veterans accessing those services [27]. The role of the peer-support specialist is to lead the patient through the VA mental health and substance use treatment system when needed, connecting and providing information about different services while encouraging them through their own story and journey and destigmatizing treatment for mental health and substance use disorders. In a cluster randomized controlled trial, patients who had a peer-support specialist in their care team had improved patient activation measures compared to patients in the control arm, who received usual care [28]. However, although improved, patient activation was still at low levels, suggesting the peer involvement was not potent enough. Other randomized studies evaluating peer support for mental health have similar limited, but positive, results justifying further investigation [29–33].

Peer support could bridge several needs of an HIV-infected veteran identified by our study participants. Peer support can provide in-person information sharing as well as emotional support and motivation around HIV treatment and overcoming HIV stigma. Challenges that may arise include role confusion over duties, integration with staff, and as well as the challenges that come with volunteer commitment such as availability and access [27]. A systematic review of peer services studied in HIV-infected populations reported positive but limited findings [26]. Alternatively, as personal connection can be quite extensive and taxing to the peer mentor [28], veterans suggested ways to obtain a similar effect through indirect measures, such as posters or videos in which a veteran with HIV shares their story. Motivational testimonies have been shown to be effective tools in behavioral change, with audio content shown more engaging than written material, thus video or recordings would be another medium in which veterans could indirectly encourage a peer with HIV [34].

## Limitations

The study was conducted in a single site, and VA study findings may not generalize to patients in other settings. The majority of participants had a VL <400 copies/mL and a CD4 cell count >200 cells/mm^3^ at most recent measurement, and a long history of HIV disease (>10 year). The majority was also an older population with 88% of participants over 50 years old. The opinions of study participants may not be representative of the opinions of non-participants with different characteristics, such as those who are younger or more recently diagnosed. Responses also may have been influenced by social desirability bias in which participants may respond in a way they think will please the interviewer or other focus group participants. It is also possible that the differing interview method (i.e. one-on-one and group interviews) affected responses. Each method has its benefits and limitations for gathering qualitative data [35]. While we attempted to include out-of-care veterans by recruiting hospitalized patients, our sample is biased towards persons interfacing with the VA system as we did not seek out of care veterans beyond those who presented themselves to the clinic or were hospitalized.

## Conclusion

The qualitative data from veteran patients living with HIV infection confirms that stigma, motivation and unmet needs are major barriers to HIV care. Positive perspectives on health, life in general and social support are facilitators. Importantly, veterans suggest practical interventions to improve retention in care including informational and normalizing brochures, videos, greater co-localization of services and in-clinic peer support. Efforts to provide positive clinic experiences, peer support and concurrent mental health care should be pursued.

## Supporting Information

S1 File Focus group and one-on-one interview guide. (DOCX)

S2 File COREQ checklist. (DOC)

S1 Table Demographic and clinical characteristics of enrolled patients (N = 107). (DOCX)

S2 Table Demographic and clinical comparisons of participating outpatients and inpatients (N = 64). (DOCX)

S3 Table Demographic and clinical characteristics of participants directly quoted in this report (N = 26). (DOCX)

S4 Table Patient-identified barriers, facilitators and interventions for attending HIV appointments. (DOCX)
