# Correction: Veterans' Perspectives on Interventions to Improve Retention in HIV Care

**DOI:** 10.1371/journal.pone.0151011

**Published:** 2016-03-03

**Authors:** Sophie G. Minick, Crystal L. Stafford, Barbara L. Kertz, Jeffery A. Cully, Melinda A. Stanley, Jessica A. Davila, Bich N. Dang, Maria C. Rodriguez-Barradas, Thomas P. Giordano

Fig 1 is incorrect. Subheadings and values are reported incorrectly. The authors have provided a corrected version here.

**Fig 1:**
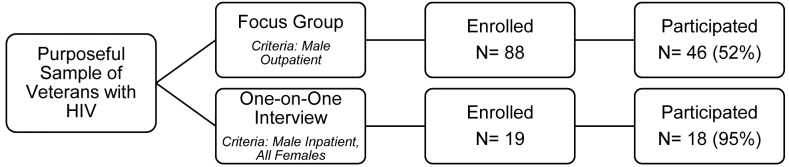
Participation flow diagram.

## References

[1] Minick SG, Stafford CL, Kertz BL, Cully JA, Stanley MA, Davila JA, et al (2016) Veterans’ Perspectives on Interventions to Improve Retention in HIV Care. PLoS ONE 11(2): e0148163 doi:10.1371/journal.pone.0148163 26829641 10.1371/journal.pone.0148163 PMC4734714

