# Supplementary material for: Veterans’ Perspectives on Interventions to Improve Retention in HIV Care
Source: PLoS One. 2016 Feb 1;11(2):e0148163. doi: 10.1371/journal.pone.0148163 (PMC4734714; doi:10.1371/journal.pone.0148163)
Supplement: S1 File — (DOCX) [file pone.0148163.s001.docx]

**S1 File. Focus group and one-on-one interview guide**

**Introduction**

- Give an overview of what should be expected for the remaining of the time during the focus group/interview (ground rules for focus group)
- Focus Group only: use Focus group sign in sheet
- Begin recording

“Thank you for agreeing to meet today. We are interested in learning about your health and continuing care through the VA Medical Center. Your participation is voluntary and your responses will be kept confidential. If you do not understand a question, please ask the research coordinator to provide clarification.

The information that you provide will be used to develop new techniques for better health care at the VA Medical Center. In order to capture everything, I will be audio recording this interview. Again, this recording will be kept confidential and your responses will not affect any services that you receive from the VA Medical Center. The audio recording will only be used to assist in writing notes after we are done talking today.”

1. **Knowledge, Attitudes, Beliefs, and expectations about HIV disease and care**
   1. What did you know about HIV disease and care when you were diagnosed?
      1. What about now?
   2. How important was it for you to see a doctor right away after you were diagnosed?
   3. Did you think you could take care of your HIV infection when you were first diagnosed?
      1. What about now?
   4. Did you think HIV treatment would help you?
      1. What about now?
   5. Did you think staying in HIV care was something you could do?
      1. What about now?
   6. Is taking care of your HIV infection something you can do easily?
      1. *PROBE: taking medications regularly*
      2. If yes, - What is makes it easy?
      3. If no, - What makes taking care of your HIV difficult?

1. **Real and perceived barriers and facilitators to entering and remaining in HIV care**
   1. What made it harder for you to seek regular medical care right after you were diagnosed?
   2. What about now, what makes it hard for you to seek regular medical care?
   3. What made it easy for you to seek regular medical care right after you were diagnosed?
   4. What about now, what makes it easy for you to seek regular medical care?
2. **Suggestions for ways to improve linkage to and retention in HIV primary care in the VA**
   1. Thinking about when you were diagnosed with HIV, what could have your doctors done or told you to make it easier for you to get into HIV care sooner?
      1. What could have other staff have done to make it easier for you to get into HIV care sooner?
      2. What could the VA have done to make it easier for you to get into HIV care sooner?
   2. What information were you lacking then that could have made a difference?
   3. Were you motivated enough, or could that have been improved?
   4. Were there any skills that you needed but didn’t have back then?
      1. *PROBE: avoiding drugs or alcohol, getting around the VA medical center*
      2. How could you have gotten those skills?

For the next questions, think about this statement: When first diagnosed with HIV, a lot of veterans make it to their first appointments but then they have difficulty staying in regular medical care

- 1. What could your doctors do or tell you to make it easier for you to stay in regular care for your HIV infection?
     1. Clarification: by “stay in regular medical care” we mean keeping all of your appointments. What could have other staff have done to make it easier for you to get into HIV care sooner?
     2. What could the VA have done to make it easier for you to get into HIV care sooner?
  2. Is there any information you feel you are lacking now that could make a difference? What?
  3. Are you motivated enough, or could that be improved? How?
  4. Are there any skills that you think you need but don’t have? avoiding drugs or alcohol, getting around the VA medical center How could you develop those skills?
  5. Is there something that other Veterans living with HIV infection could have done or told you that would have made a difference?
     1. What? Could you give us some details.
     2. *PROBE: Should changes be permanent or temporary?*
     3. *PROBE: How long should the extra support last?*
  6. In what ways could the information get to you?

1. **Information on HIV care through social media techniques, messages, messengers and media**
   1. If you were designing an ad to promote Veterans living with HIV to stay in regular care, what would it say?
   2. Who would be in the ads (patients, doctors, celebrities, others)?
   3. When is the best time to receive information regarding HIV care?
      1. *PROBE: at time of diagnosis, ongoing, at appointments and hospital visits, time of day*
   4. How could the ads reach the Veterans?
      1. *PROBE: commercials, newspaper, other print media*
