# Supplementary material for: Veterans’ Perspectives on Interventions to Improve Retention in HIV Care
Source: PLoS One. 2016 Feb 1;11(2):e0148163. doi: 10.1371/journal.pone.0148163 (PMC4734714; doi:10.1371/journal.pone.0148163)
Supplement: S2 File — (DOC) [file pone.0148163.s002.doc]

**S2_File**

**On Reflexivity**

Author Crystal L. Stafford interviewed patients, while co-author Barbara L. Kertz took notes in the room during interviews. Three authors, Crystal L. Stafford, Barbara L. Kertz, and Sophie G. Minick, coded the data gathered. Their information for reflexivity purposes is described below. Bias control was maintained through reflexive debriefings throughout analysis.

Crystal L. Stafford, DrPH, recruited patients and conducted every interview and focus group for this project. At the time of the study she was a public health researcher at the VA in Health Services Research, interested in health care delivery and health care outcomes. Her past experience includes qualitative projects for her doctorate degree and extensive research training. She did not have a relationship with participants established prior to the interview and personal characteristics comprised of her introduction as interested in health care outcomes particularly for improving HIV care at the VA. She was involved in coding and analyzing the data after the interviews.

Barbara L. Kertz, BS, participated in the interviews through taking field notes in the background, coding the data and analyzing the data. She did not interact with subjects during the interview. At the time of the study she was a research coordinator in the VA Infectious Disease group. She was introduced to participants as a researcher interested in the barriers and facilitators of HIV care. Her past experience includes several qualitative studies and appropriate research training.

Sophie G. Minick, BA, coded and analyzed the data after the interviews were completed. She did not have any contact with the participants. She is a research coordinator for Baylor College of Medicine. Her past experience includes qualitative studies during her time as an undergraduate. She has been trained in qualitative analysis, research conduct, statistical analysis, and use of Atlas ti.

**Consolidated criteria for reporting qualitative studies (COREQ): 32-item checklist**

Developed from:

Tong A, Sainsbury P, Craig J. Consolidated criteria for reporting qualitative research (COREQ): a 32-item checklist for interviews and focus groups. *International Journal for Quality in Health Care*. 2007. Volume 19, Number 6: pp. 349 – 357

| **No. Item** | **Guide questions/description** | **Report** |
| --- | --- | --- |
| **Domain 1: Research team and reﬂexivity** |  |  |
| *Personal Characteristics* |  |  |
| 1. Interviewer/ facilitator | Which author/s conducted the interview or focus group? | CLS and BLK (pg. 6) |
| 2. Credentials | What were the researcher’s credentials? E.g. PhD, MD | CLS: DrPH  BLK: BS  SGM: BA (pg. 6) |
| 3. Occupation | What was their occupation at the time of the study? | CLS: Research Coordinator  BLK: Research Coordinator  SGM: Research Coordinator (pg. 6) |
| 4. Gender | Was the researcher male or female? | All female (pg. 6) |
| 5. Experience and training | What experience or training did the researcher have? | CLS: qualitative projects for her doctorate degree and extensive research training.  BLK: several qualitative studies and appropriate research training  SGM: own qualitative studies, trained in qualitative analysis, research conduct, statistical analysis, Atlas ti |
| *Relationship with participants* |  |  |
| 6. Relationship established | Was a relationship established prior to study commencement? | Relationship was not established prior to interviews |
| 7. Participant knowledge of the interviewer | What did the participants know about the researcher? e.g. personal goals, reasons for doing the research | Personal interest in research and reasons for doing it were described prior to interviews during recruitment if asked, but introduced as a public health researcher. Interests are listed with introductions on (pg. 6) Reasons for research was described in detail at times of recruitment and while providing informed consent. |
| 8. Interviewer characteristics | What characteristics were reported about the inter viewer/facilitator? e.g. Bias, assumptions, reasons and interests in the research topic | Personal interviewer characteristics were not discussed with the participants but the purpose of the study was discussed in detail at time of recruitment in order to obtain written informed consent. (pg. 4) |
| **Domain 2: study design** |  |  |
| *Theoretical framework* |  |  |
| 9. Methodological orientation and Theory | What methodological orientation was stated to underpin the study? e.g. grounded theory, discourse analysis, ethnography, phenomenology, content analysis | Content Analysis (pg. 6) |
| *Participant selection* |  |  |
| 10. Sampling | How were participants selected? e.g. purposive, convenience, consecutive, snowball | Purposeful sampling was used to identify a sample of patients with HIV infection that included men and women and persons with experience of being not retained and retained in care. (pg. 4) |
| 11. Method of approach | How were participants approached? e.g. face-to-face, telephone, mail, email | Patients were approached face-to-face when they presented to HIV clinic (pg. 4,5) |
| 12. Sample size | How many participants were in the study? | 107 enrolled, 46 participated (pg. 8) |
| 13. Non-participation | How many people refused to participate or dropped out? Reasons? | 61 people did not participate. Reasons were not sought (pg. 8) |
| *Setting* |  |  |
| 14. Setting of data collection | Where was the data collected? e.g. home, clinic, workplace | Houston’s Michael E. DeBakey VA Medical Center (pg. 5) |
| 15. Presence of non-participants | Was anyone else present besides the participants and researchers? | No (pg. 6) |
| 16. Description of sample | What are the important characteristics of the sample? e.g. demographic data, date | Demographic data, relevant medical records reviewed for first CD4 cell count and HIV viral load and appointment adherence data to measure retention in care (pg. 5, 8) |
| *Data collection* |  |  |
| 17. Interview guide | Were questions, prompts, guides provided by the authors? Was it pilot tested? | Interview approach addressed in methods (pg. 5) and also attached as supplementary information see S1 File (pg. 26). It was not pilot tested. |
| 18. Repeat interviews | Were repeat interviews carried out? If yes, how many? | No, interviews were only conducted once |
| 19. Audio/visual recording | Did the research use audio or visual recording to collect the data? | Yes audio recording was used to collect data for transcription (pg. 6) |
| 20. Field notes | Were ﬁeld notes made during and/or after the inter view or focus group? | Yes , BLK took field notes during the sessions. (pg. 6) |
| 21. Duration | What was the duration of the interviews or focus group? | Up to 60 minutes (pg. 6 ) |
| 22. Data saturation | Was data saturation discussed? | Yes, data saturation was discussed among the research coordinators and with the PI. Recruitment ceased once saturation in each stratum was reached. (pg6) |
| 23. Transcripts returned | Were transcripts returned to participants for comment and/or correction? | No |
| **Domain 3: analysis and ﬁndings** |  |  |
| *Data analysis* |  |  |
| 24. Number of data coders | How many data coders coded the data? | 3 coders ( pg7) |
| 25. Description of the coding tree | Did authors provide a description of the coding tree? | No |
| 26. Derivation of themes | Were themes identiﬁed in advance or derived from the data? | 3 categories were pre-specified: barriers, facilitators and interventions for HIV care); codes within the core categories were not pre-specified but driven by the data (pg. 6) |
| 27. Software | What software, if applicable, was used to manage the data? | Atlas ti version 6.2 (pg. 6) was used to manage the qualitative data and SAS was used for demographic data. |
| 28. Participant checking | Did participants provide feedback on the ﬁndings? | No |
| *Reporting* |  |  |
| 29. Quotations presented | Were participant quotations presented to illustrate the themes/ﬁndings? Was each quotation identiﬁed? e.g. participant number | Yes, quotations were identified with participant numbers connected to demographic details to provide the relevant context behind each quote (see S 1 Table, pg. 26) |
| 30. Data and ﬁndings consistent | Was there consistency between the data presented and the ﬁndings? | Yes |
| 31. Clarity of major themes | Were major themes clearly presented in the ﬁndings? | Yes |
| 32. Clarity of minor themes | Is there a description of diverse cases or discussion of minor themes? | Yes |
