# Supplementary material for: Veterans’ Perspectives on Interventions to Improve Retention in HIV Care
Source: PLoS One. 2016 Feb 1;11(2):e0148163. doi: 10.1371/journal.pone.0148163 (PMC4734714; doi:10.1371/journal.pone.0148163)
Supplement: S1 Table — (DOCX) [file pone.0148163.s003.docx]

|  | **Non Participants**  **(n =43)** | **Participants**  **(n=64)** | **P-Value** |
| --- | --- | --- | --- |
| *Age* |  |  |  |
| <40 | 5 (12%) | 2 (3%) | 0.10 |
| 40-49 | 7 (16%) | 6 (9%) |  |
| 50+ | 31 (72%) | 56 (88%) |  |
| *Gender* |  |  |  |
| Male | 42 (98%) | 58 (91%) | 0.15 |
| Female | 1 (2%) | 6 (10%) |  |
| *Race/ethnicity* |  |  |  |
| Black (non-Hispanic) | 35 (81%) | 43 (67%) | 0.22 |
| Hispanic | 2 (5%) | 8 (13%) |  |
| White (non-Hispanic) | 6 (14%) | 13 (20%) |  |
| *Sexual orientation* |  |  |  |
| Gay or Lesbian | 9 (21%) | 21 (33%) | 0.31 |
| Bisexual | 4 (9%) | 6 (9%) |  |
| Straight | 30 (70%) | 35 (55%) |  |
| Unsure or in transition | 0 (0%) | 2 (3%) |  |
| *Years from diagnosis* |  |  |  |
| ≤5 years | 5 (12%) | 5 (8%) | 0.21 |
| 6 -10 years | 9 (21%) | 7 (11%) |  |
| 11- 20 years | 18 (42%) | 24 (38%) |  |
| >20 years | 11 (26%) | 28 (44%) |  |
| *Retention in care** |  |  |  |
| *No gap in care ≥ 180 days in last two years* |  |  |  |
| Not retained | 27 (63%) | 39 (63%) | 0.99 |
| Retained | 16 (37%) | 23 (37%) |  |
| *Constancy in care in last 2 years (≥ 1 visit in each 6-month block)* |  |  |  |
| Not retained | 20 (48%) | 27 (44%) | 0.74 |
| Retained | 22 (52%) | 34 (56%) |  |
| *Constancy in care in last 1 year (≥ 1 visit in 3 or 4 quarter-years)* |  |  |  |
| Not retained | 16 (38%) | 24 (39%) | 0.95 |
| Retained | 26 (62%) | 38 (61%) |  |
| *Not retained by any of the 3 definitions above* |  |  |  |
| Not retained | 29 (69%) | 44 (72%) | 0.74 |
| Retained | 13 (31%) | 17 (28%) |  |
| *First HIV viral load*** |  |  |  |
| ≤ 400 c/mL | 12 (29%) | 16 (25%) | 0.68 |
| > 400 c/mL | 30 (71%) | 48 (75%) |  |
| *First CD4 cell count* |  |  |  |
| <200 cells/mm3 | 12 (28%) | 15 (23%) | 0.62 |
| 200- 500 cells/mm3 | 20 (47%) | 27 (42%) |  |
| > 500 cells/mm3 | 11 (26%) | 22 (34%) |  |
| *Last HIV viral load* |  |  |  |
| ≤ 400 c/mL | 37 (86%) | 51 (80%) | 0.40 |
| > 400 c/mL | 6 (14%) | 13 (20%) |  |
| *Last CD4 cell count* |  |  |  |
| < 200 cells/mm3 | 7 (16%) | 10 (16%) | 0.39 |
| 200 – 500 cells/mm3 | 14 (33%) | 29 (45%) |  |
| > 500 cells/mm3 | 22 (51%) | 25 (39%) |  |

*Veterans excluded from retention measures include: two Veterans from the gap in care measure due to only having one visit in time period; four Veterans from the two year retention measure due to either newly diagnosed or new to the VA within 2 years; three Veterans from the one year retention measure due to either diagnosed or new to VA within one year.

** One non-participant’s initial viral load was missing.
