# Supplementary material for: Veterans’ Perspectives on Interventions to Improve Retention in HIV Care
Source: PLoS One. 2016 Feb 1;11(2):e0148163. doi: 10.1371/journal.pone.0148163 (PMC4734714; doi:10.1371/journal.pone.0148163)
Supplement: S2 Table — (DOCX) [file pone.0148163.s004.docx]

**S2 Table. Demographic and clinical comparisons of participating outpatients and inpatients (N=64).**

|  | **Hospitalized (n=14)** | **Non-Hospitalized (n=50)** | **P-Value** |
| --- | --- | --- | --- |
| *Age* |  |  |  |
| <40 | 0 (0%) | 2 (4%) | 0.60 |
| 40-49 | 2 (14%) | 4 (8%) |  |
| 50+ | 12 (86%) | 44 (88%) |  |
| *Gender* |  |  |  |
| Male | 12 (86%) | 46 (92%) | 0.48 |
| Female | 2 (14%) | 4 (8%) |  |
| *Race/ethnicity* |  |  |  |
| Black | 12 (86%) | 31 (62%) | 0.24 |
| Hispanic | 1 (7%) | 7 (14%) |  |
| White | 1 (7%) | 12 (24%) |  |
| *Sexual Orientation* |  |  |  |
| Gay or Lesbian | 3 (21%) | 18 (36%) | 0.57 |
| Bisexual | 1 (7%) | 5 (10%) |  |
| Straight | 9 (64%) | 26 (52%) |  |
| Unsure or in transition | 1 (7%) | 1 (2%) |  |
| *Years from Dx* |  |  |  |
| ≤5 years | 1 (7%) | 4 (8%) | 0.94 |
| 6 -10 years | 1 (7%) | 6 (12%) |  |
| 11- 20 years | 6 (43%) | 18 (36%) |  |
| >20 years | 6 (43%) | 22 (44%) |  |
| *Retention in care** |  |  |  |
| *No gap in care ≥ 180 days in last two years* |  |  |  |
| Not retained | 9 (69%) | 30 (61%) | 0.60 |
| Retained | 4 (31%) | 19 (39%) |  |
| *Constancy in care in last 2 years (≥ 1 visit in each 6-month block)* |  |  |  |
| Not retained | 8 (62%) | 19 (40%) | 0.16 |
| Retained | 5 (38%) | 29 (60%) |  |
| *Constancy in care in last 1 year (≥ 1 visit in 3 or 4 quarter-years)* |  |  |  |
| Not retained | 9 (69%) | 15 (31%) | 0.01 |
| Retained | 4 (31%) | 34 (69%) |  |
| *Not retained by any of the 3 definitions above* |  |  |  |
| Not retained | 11 (85%) | 33 (69%) | 0.26 |
| Retained | 2 (15%) | 15 (31%) |  |
| *First HIV Viral Load result [copies/mL]* |  |  |  |
| ≤ 400 | 2 (14%) | 14 (28%) | 0.30 |
| > 400 | 12 (86%) | 36 (72%) |  |
| *First CD4 cell count result [cells/mm^3^]* |  |  |  |
| <200 | 3 (21%) | 12 (24%) | 0.79 |
| 200 – 500 | 7 (50%) | 20 (40%) |  |
| > 500 | 4 (29%) | 18 (36%) |  |
| *Current HIV Viral Load result [copies/mL]* |  |  |  |
| ≤ 400 | 9 (64%) | 42 (84%) | 0.11 |
| > 400 | 5 (36%) | 8 (16%) |  |
| *Current CD4 cell count result [cells/mm^3^]* |  |  |  |
| < 200 | 7 (50%) | 3 (6%) | <0.0001 |
| 200 – 500 | 7 (50%) | 22 (44%) |  |
| > 500 | 0 (0%) | 25 (50%) |  |

*Veterans excluded from retention measures include: two Veterans from the gap in care measure due to only having one visit in time period; three Veterans from the two year retention measure due to either newly diagnosed or new to the VA within two years; two Veterans from the one year retention measure due to either diagnosed or new to VA within one year.
