# Supplementary material for: Veterans’ Perspectives on Interventions to Improve Retention in HIV Care
Source: PLoS One. 2016 Feb 1;11(2):e0148163. doi: 10.1371/journal.pone.0148163 (PMC4734714; doi:10.1371/journal.pone.0148163)
Supplement: S3 Table — (DOCX) [file pone.0148163.s005.docx]

| Participant number | Age | Sex | Race/ Ethnicity | Self-Reported Sexual Orientation | Years From HIV Diagnosis | Current CD4 cell count (cells/mm3) | Current HIV Viral Load <400  copies/mL | Not retained in care in last 2 years by any of the 3 retention measures* |
| --- | --- | --- | --- | --- | --- | --- | --- | --- |
| 001 | 50+ | Male | Black | Heterosexual | >20 | >500 | Yes | Yes |
| 002 | 50+ | Male | Black | Homosexual | >20 | 200-500 | Yes | Yes |
| 003 | 50+ | Female | Black | Heterosexual | 6-10 | 200-500 | Yes | Yes |
| 004 | 50+ | Female | Black | Heterosexual | 6-10 | >500 | Yes | Yes |
| 005 | 50+ | Female | Black | Unsure/  In-transition | 11-20 | <200 | Yes | Yes |
| 006 | 40-49 | Male | White | Homosexual | 11-20 | >500 | Yes | Yes |
| 007 | 50+ | Male | Black | Heterosexual | ≤5 | 200-500 | Yes | No |
| 008 | 50+ | Male | Black | Unsure/  In-transition | >20 | 200-500 | Yes | Yes |
| 009 | 40-49 | Male | Black | Bisexual | >20 | 200-500 | Yes | Yes |
| 010 | 50+ | Male | Black | Heterosexual | 11-20 | 200-500 | Yes | Yes |
| 011 | 50+ | Male | Hispanic | Homosexual | ≤5 | 200-500 | Yes | Yes |
| 012 | <40 | Male | Black | Heterosexual | 11-20 | 200-500 | No | Yes |
| 013 | 50+ | Male | White | Bisexual | 11-20 | >500 | Yes | No |
| 014 | 50+ | Male | White | Heterosexual | >20 | 200-500 | Yes | Yes |
| 015 | 50+ | Male | White | Homosexual | >20 | >500 | Yes | Yes |
| 016 | 50+ | Male | Black | Heterosexual | 11-20 | 200-500 | No | No |
| 017 | <40 | Male | White | Homosexual | 6-10 | >500 | Yes | No |
| 018 | 50+ | Male | Black | Heterosexual | 11-20 | 200-500 | Yes | Yes |
| 019 | 50+ | Male | White | Bisexual | 6-10 | >500 | Yes | Yes |
| 020 | 50+ | Male | Black | Heterosexual | >20 | >500 | No | Yes |
| 021 | 40-49 | Female | Black | Heterosexual | 6-10 | >500 | Yes | Yes |
| 022 | 40-49 | Male | White | Homosexual | >20 | <200 | No | Yes |
| 023 | 50+ | Female | White | Heterosexual | >20 | <200 | Yes | Yes |
| 024 | 50+ | Male | Black | Bisexual | 11-20 | 200-500 | Yes | No |
| 025 | 50+ | Male | White | Homosexual | >20 | >500 | Yes | Yes |
| 026 | 50+ | Male | Black | Heterosexual | >20 | >500 | Yes | No |

*Defined as meeting at least one of the following recognized measures for not being retained in care: 1) a gap between appointments spanning longer than 180 days in the last 2 years, 2) attending a visit in each of the four 6-month blocks throughout the last two years, and 3) attending a visit in three or four of the quarter-years in the last year.
