# Supplementary material for: Veterans’ Perspectives on Interventions to Improve Retention in HIV Care
Source: PLoS One. 2016 Feb 1;11(2):e0148163. doi: 10.1371/journal.pone.0148163 (PMC4734714; doi:10.1371/journal.pone.0148163)
Supplement: S4 Table — (DOCX) [file pone.0148163.s006.docx]

**S4 Table. Patient-identified barriers, facilitators and interventions for attending HIV appointments.**

|  | **Themes** | **Subthemes** |
| --- | --- | --- |
| **Barriers – What is preventing you from regularly attending HIV appointments?** | **My Self –** *“I am my worst enemy.”* Attitudes/perspective | Low self-worth |
|  |  | Hopelessness |
|  |  | Wrong mindset from inaccurate knowledge |
|  |  | Fatigue of ongoing illness |
|  |  | Feeling healthy – care unnecessary |
|  |  | Perceived Stigma |
|  |  | Careless lifestyle |
|  | **My Needs –** *“I need more help than I can get on my own.”* Lack of resources to address needs | Options and services not known (not utilizing available services) |
|  |  | Mental health issues |
|  |  | Substance Use |
|  |  | Financial burden from co-pay and time off work |
|  |  | Work schedule |
|  |  | Lack of social support |
|  |  | Lack of knowledge of support systems |
|  | **My Clinic –** *“Hurry up and wait.”* Difficult system and VA characteristics | Poor clinic experience |
|  |  | Long wait times |
|  |  | Parking |
|  |  | Lack of HIV display |
|  |  | Difficulty getting into programs |
|  |  | Unpleasant doctor or staff interactions |
| **Facilitators – What is helping you?** | **My Desire –** *“I want to stay alive and healthy.”* Motivated to value own health | Transformative moment: Desire to live |
|  |  | Positive mindset: Living to live not to die |
|  |  | Taking responsibility: valuing and tracking own health |
|  |  | Being there for family: children and grandchildren give reason to live |
|  | **My Perspective –** *“I’m not dying of AIDS I’m living with HIV.”* Shown health is achievable | Role models- Celebrities with HIV |
|  |  | Patient models – others’ testimonies |
|  |  | Trust in doctor |
|  | **My Community –** *“Everybody needs a support system.”* Ongoing social support | Caring atmosphere at the VA |
|  |  | Good relationship with doctor |
|  |  | Social support |
|  | **My Tools –** *“The system’s great.”* Equipped to meet needs | Case management to connect services |
|  |  | Financial assistance |
|  |  | Phone call and mail reminders |
|  |  | Peer support for advice and solution |
|  |  | Support group |
| **Interventions – What should be addressed to help veterans receive HIV care?** | **Our Services** *–“Let’s make you a map and get you there.”* Comprehensively addressing needs | Support for unmet needs – drug rehabilitation, mental health, finance, housing, transportation |
|  |  | Service information lounge |
|  |  | Case manager – take time for simple instructions on what to do and how to do it |
|  |  | Provide Incentives – financial, interpersonal, gaining skills |
|  | **Our Knowledge** – *“A lot of people just don’t know about HIV. They think they know but they really don’t.”* Awareness through education | Educational courses about HIV |
|  |  | Educate the public to raise awareness and decrease stigma |
|  |  | More information in clinic: brochure, pamphlets bulletins |
|  |  | Navigation of the VA- Video: understand the specific process |
|  | **Our Support –** *“I’m not alone… you know it's another soldier.”* Continuously feed the motivation | Testimonial video : patients living with HIV |
|  |  | Celebrity role models coming forth |
|  |  | Mutually beneficial peer relationship |
|  |  | Walking together: share experience via buddy system |
|  |  | Walking ahead: Peer navigation |
